# Respiratory pathogens detected in children aged <5 years hospitalized with severe respiratory illness, South Africa, 2017

**DOI:** 10.3389/fped.2025.1498197

**Published:** 2025-06-27

**Authors:** Malefu Moleleki, Cayla Reddy, Kedibone Ndlangisa, Mignon du Plessis, Orienka Hellferscee, Omphe Mekgoe, Sibongile Walaza, Stefano Tempia, Cheryl Cohen, Anne von Gottberg, Nicole Wolter

**Affiliations:** ^1^ Centre for Respiratory Diseases and Meningitis, National Institute for Communicable Diseases of the National Health Laboratory Service, Johannesburg, South Africa; ^2^ Department of Clinical Microbiology and Infectious Diseases, School of Pathology, Faculty of Health Sciences, University of the Witwatersrand, Johannesburg, South Africa; ^3^ Department of Medical Virology, School of Pathology, Faculty of Health Sciences, University of the Witwatersrand, Johannesburg, South Africa; ^4^ Department of Paediatrics, Faculty of Health Sciences, University of the Witwatersrand, Johannesburg, South Africa; ^5^ School of Public Health, Faculty of Health Sciences, University of the Witwatersrand, Johannesburg, South Africa; ^6^ MassGenics, Duluth, GA, United States

**Keywords:** severe respiratory illness, community-acquired pneumonia, TaqMan Array Card, childhood pneumonia, South Africa, respiratory pathogens, RSV

## Abstract

**Introduction:**

The burden of morbidity and mortality of severe respiratory illness (SRI) remains disproportionately high among young children, and in low-and middle-income countries. We used a multi-pathogen respiratory PCR assay to detect pathogens in children aged <5 years hospitalized with SRI.

**Methods:**

Prospective syndromic surveillance for SRI was performed at two sentinel hospitals in South Africa between January and December 2017. Nasopharyngeal aspirates and sputa were collected and tested using a real-time polymerase chain reaction based TaqMan Array Card (TAC) for the detection of 21 respiratory pathogens. Pathogen detection was compared by age group using the chi-squared test and seasonal frequency analysed.

**Results:**

From January through December 2017, 361 children were enrolled and of these, 198 cases with sufficient specimen volume were included in this study. Overall, 189/198 (95%) of the children tested positive for at least one pathogen. Common viruses identified included rhinovirus (65/198; 33%), respiratory syncytial virus (RSV) (54/198; 27%), adenovirus (34/198; 17%), and enterovirus (28/198; 14%). Common bacteria detected included *Haemophilus influenzae* (121/198; 61%), *Streptococcus pneumoniae* (114/198; 58%), *Klebsiella pneumoniae* (61/198; 31%), *Staphylococcus aureus* (52/198; 26%), and *Acinetobacter baumannii* (27/198; 14%).

**Discussion:**

Bacterial detections were high in our study driven by the high detection of *S. pneumoniae* and *H. influenzae*. Co-detections of pathogens were common and require clinical evaluation to determine their relevance in clinical management. Further, given the high prevalence of RSV amongst children hospitalized with SRI, there is an urgent need for continued efforts towards access to maternal RSV vaccines and therapeutic interventions such as monoclonal antibodies particularly in low- and middle-income countries which experience the highest burden of RSV-associated disease.

## 1 Introduction

The burden of morbidity and mortality of severe respiratory illness (SRI) remains disproportionately high among children aged <5 years globally and is highest in the first year of life (1, 2). In this age group, respiratory viruses, such as respiratory syncytial virus (RSV) and influenza are commonly responsible for the majority of infections however, bacterial infections, such as with *Streptococcus pneumoniae* and non-typeable *Haemophilus influenzae* may occur following an acute viral infection due to mucosal invasion or aspiration of nasopharyngeal bacteria and are associated with more severe outcomes of SRI (3–7).

The South African Thoracic Society's SRI treatment guidelines recommend that all children with signs of severe pneumonia be given empiric antibiotic therapy for at least five days, the escalation, de-escalation or discontinuation of which should be advised by the patient's response to therapy and/or microbiology results (8). Polymerase chain reaction (PCR) is standard for the detection of respiratory viruses and atypical pneumonia-causing bacteria, however, in South Africa, PCR is not routinely performed as part of the diagnostic workflow in SRI as treatment is usually empiric. Accurate diagnosis of the causative pathogen is critical for optimizing effective patient management decisions, such as the need for antibiotic or antiviral prescriptions or to inform empiric therapy.

We used a multi-pathogen real-time PCR-based respiratory panel to detect viruses and bacteria in children aged <5 years hospitalized with SRI at two sentinel hospitals in South Africa.

## 2 Materials and methods

### 2.1 Study population

Prospective hospital-based syndromic surveillance for SRI in all ages was initiated in 2009 in South Africa to describe the aetiology of and risk factors for community-acquired pneumonia. Children aged <5 years hospitalized with SRI and enrolled at two sentinel hospitals in the North-West and Mpumalanga Provinces between January and December 2017 were included in the current study. In-hospital outcome (discharge or death) was recorded.

### 2.2 Case definition

A case of SRI was defined as any hospitalized child, regardless of symptom duration, in age-defined categories as follows: any child aged 2 days to <3 months with diagnosis of suspected sepsis or physician diagnosed lower respiratory tract infection (LRTI) irrespective of signs and symptoms and, any child ≥3 months to <5 years with physician-diagnosed acute LRTI including bronchiolitis, pneumonia, bronchitis and pleural effusion (9). Surveillance officers administered a questionnaire with demographic information and obtained clinical information from medical records.

### 2.3 Specimen collection

Nasopharyngeal aspirates (NPA) were collected from all children and induced sputum specimens were collected from individuals aged ≥3 months. If induced sputum collection was contraindicated or was not advised by the attending clinician and a patient was able to expectorate, expectorated sputum was collected. Specimens were transported to the National Institute for Communicable Diseases, Johannesburg, South Africa, for testing and thereafter stored at −80°C.

### 2.4 Laboratory testing

From January through December 2017, 361 children were enrolled and of these, 198 cases with sufficient specimen volume (200 µl) were included in this study. Total nucleic acids (TNA) were extracted from 200 µl of specimen using the MagNA Pure Compact instrument (Roche Diagnostics, Mannheim, Germany) with Total Nucleic Acid Isolation Kit I according to the manufacturer's instructions and thereafter stored at −20°C.

Real-time PCR was carried out for the detection of 21 viruses and bacteria using a custom-made TaqMan Array Card (TAC) developed from previously published assays (10–12). Pathogens tested, in duplicate, included *Mycobacterium tuberculosis*, *Staphylococcus aureus*, *S. pneumoniae, H. influenzae,* Group B streptococci (GBS), *Mycoplasma pneumoniae, Bordetella* spp., *Legionella* spp., *Acinetobacter baumannii*, *Klebsiella pneumoniae*, *Pseudomonas aeruginosa,* adenovirus, human metapneumovirus (hMPV), enterovirus, influenza types A and B, parainfluenza virus types 1–3, RSV, and rhinovirus. TACs included two controls; the internal positive control (IPC) for monitoring of the real-time PCR reaction and a human *RNAseP* gene control for monitoring of specimen quality.

TAC assays were performed using 50 µl qScript XLT 1-step RT-qPCR Toughmix (Quantabio, Beverly, Massachusetts, USA) and 50 µl of TNA extract. TAC PCR was carried out on the Applied Biosystems ViiA7 and QuantStudio 7 Real Time PCR systems (Life Technologies, New York, USA) using the following cycling conditions: 45°C for 10 min, 94°C for 10 min, 45 cycles of 94°C for 30 s and 60°C for 1 min. A no template control (NTC) and positive control consisting of combined RNA transcripts generated as previously described by Kodani et al. were included on each TAC (13). A positive result was recorded if amplification occurred in at least one of the duplicate reactions with cycle threshold (Ct) <40.

### 2.5 Statistical analysis

Stata 14 (Stata Corporation, College Station, TX) was used for statistical analysis. McNemar's *χ*^2^ test or Fischer's exact test were used, where appropriate, to compare categorical variables (*p*-value <0.05 was considered statistically significant). Pathogen prevalence was stratified by season in which December—February were classified as summer, March—May as autumn, June—August as winter, and September—November as spring (14).

## 3 Results

### 3.1 Study population

From January through December 2017, 361 patients aged <5 years were enrolled in the SRI surveillance program, of which 228 (63%) were diagnosed with LRTI at discharge, 36 (10%) with bronchiolitis while 141 (39%) were diagnosed with other illnesses including bronchitis, tuberculosis, and sepsis. Of the 361 cases enrolled, 198 (55%) were included in this study if at least one respiratory specimen (NPA and/or sputum) were available for testing (Table 1).The median age of patients tested on TAC was 10 months [interquartile range (IQR) 4–21 months], 57% (113/198) were aged <1 year and 89% (177/195) were HIV-uninfected. Antibiotic therapy was initiated in 96% (191/196) of the patients at admission and the in-hospital case-fatality proportion was 1% (2/192).

**Table 1:** Demographic and clinical characteristics of children aged <5 years hospitalized with severe respiratory illness (SRI) at two sentinel sites in South Africa, January–December 2017.

Characteristic	All patients (*N* = 361)	Tested on TAC (*N* = 198)	*p*-value	Not tested on TAC (*N* = 163)
	*n* (%)	*n* (%)		*n* (%)
Gender				
Female	171 (47)	88 (44)	0.535	83 (51)
Age in months				
Median age (IQR)	8 (4–17)	10 (4–21)	N/A	8 (4–14)
Age group in years				
<1	232 (64)	113 (57)	0.102	119 (73)
1–4	129 (36)	85 (43)		44 (27)
Site province				
Mpumalanga	163 (45)	99 (50)	0.288	64 (39)
North-West	198 (55)	99 (50)		99 (61)
Season of enrolment*				
Summer	78 (22)	49 (25)	**<0**.**001**	29 (18)
Autumn	105 (29)	75 (38)		30 (18)
Winter	79 (22)	54 (27)		25 (15)
Spring	99 (27)	20 (10)		79 (48)
Antibiotic therapy				
Yes	340 (94)	191 (96)	0.368	149 (91)
No	18 (5)	5 (3)		13 (8)
Unknown	3 (1)	2 (1)		1 (1)
Outcome				
Discharge	345 (96)	190 (96)	0.930	155 (95)
Death	5 (1)	2 (1)		3 (2)
Unknown	11 (3)	6 (3)		5 (3)
HIV status				
ILWH	30 (8)	18 (9)	0.815	12 (7)
HIV uninfected	323 (89)	177 (89)		146 (90)
Unknown	8 (2)	3 (2)		5 (3)
PCV status				
Fully vaccinated	287 (80)	160 (80)	0.964	127 (78)
Unvaccinated	30 (8)	14 (7)		16 (10)
N/A (aged <6 weeks)	20 (6)	11 (6)		9 (6)
Unknown	24 (7)	13 (7)		11 (7)

* Seasons were defined as follows: summer, Dec/Jan/Feb; autumn, Mar/Apr/May; winter, Jun/Jul/Aug; spring, Sep/Oct/Nov. ILWH, individuals living with HIV. PCV, pneumococcal conjugate vaccine. *P* < 0.05 considered statistically significant when comparing characteristics between total patients enrolled and those tested using TAC. N/A, non-applicable.

### 3.2 Prevalence of respiratory pathogens

Among the 198 patients tested, 183 (92%) had a NPA tested and 44 (22%) had sputum tested. Among these, 154 (78%) had a NPA only tested, 15 (8%) sputum only and 29 (15%) had both sputum and NPA tested. Overall, 189/198 (95%) of children tested positive for at least one respiratory pathogen: 89% (177/198) for ≥1 bacterial pathogen and 79% (156/198) for ≥1 viral pathogen. Common viruses identified included rhinovirus (65/198; 33%), RSV (54/198; 27%), adenovirus (34/198; 17%), and enterovirus (28/198; 14%). Common bacteria detected included *H. influenzae* (121/198; 61%), *S. pneumoniae* (114/198; 58%), *K. pneumoniae* (61/198; 31%), *S. aureus* (52/198; 26%), and *A. baumannii* (27/198; 14%) (Table 2). Of the patients in which both sputum and NPA specimens were available for testing (*n* = 29), there was a high congruence in the detection of *H. influenzae* (91%) and *S. pneumoniae* (82%) between specimen types (Supplementary Table S1). On the other hand, congruence was moderate to low for the detection of *K. pneumoniae* (57%), *S. aureus* (40%), and *A. baumannii* (20%), which were more commonly detected in NPA compared to sputum.

**Table 2:** Pathogens detected in children aged <5 years hospitalized at two sentinel sites with severe respiratory illness by specimen type, South Africa, January–December 2017.

Pathogen	Any respiratory specimen; *n* (%) (*N* = 198)	NPA; *n* (%) (*N* = 183)	Sputum; *n* (%) (*N* = 44)	*p*-value
Bacteria				
*H. influenzae*	121 (61)	109 (60)	33 (75)	0.082
*S. pneumoniae*	114 (58)	102 (56)	31 (70)	0.089
*K. pneumoniae*	61 (31)	55 (30)	10 (23)	0.361
*S. aureus*	52 (26)	45 (25)	9 (20)	0.694
*A. baumannii*	27 (14)	21 (11)	7 (16)	0.445
*P. aeruginosa*	18 (9)	14 (8)	4 (9)	0.530
GBS	11 (6)	9 (5)	2 (5)	1.000
*M. tuberculosis*	5 (3)	2 (1)	3 (7)	**0**.**051**
*Bordetella* spp.	3 (2)	2 (1)	1 (2)	0.478
*M. pneumoniae*	2 (1)	1 (1)	1 (2)	0.351
*Legionella* spp.	1 (1)	1 (1)	0	1.000
Viruses				
Rhinovirus	65 (33)	60 (33)	14 (32)	1.000
RSV	54 (27)	50 (27)	12 (27)	1.000
Adenovirus	34 (17)	20 (11)	16 (36)	**<0**.**001**
Enterovirus	28 (14)	27 (15)	5 (11)	0.809
Parainfluenza 2	11 (6)	11 (6)	1 (2)	0.469
hMPV	10 (5)	10 (5)	2 (5)	1.000
Influenza A	10 (5)	10 (5)	1 (2)	0.696
Parainfluenza 3	6 (3)	4 (2)	3 (7)	0.134
Parainfluenza 1	5 (3)	4 (2)	2 (5)	0.329
Influenza B	5 (3)	2 (1)	2 (5)	0.170

NPA, nasopharyngeal aspirate. GBS, group B streptococci; RSV, respiratory syncytial virus; hMPV, human metapneumovirus; NT, not tested. *P* < 0.05 considered statistically significant when comparing pathogen detection between sputum and NPA.

**Figure 1:**
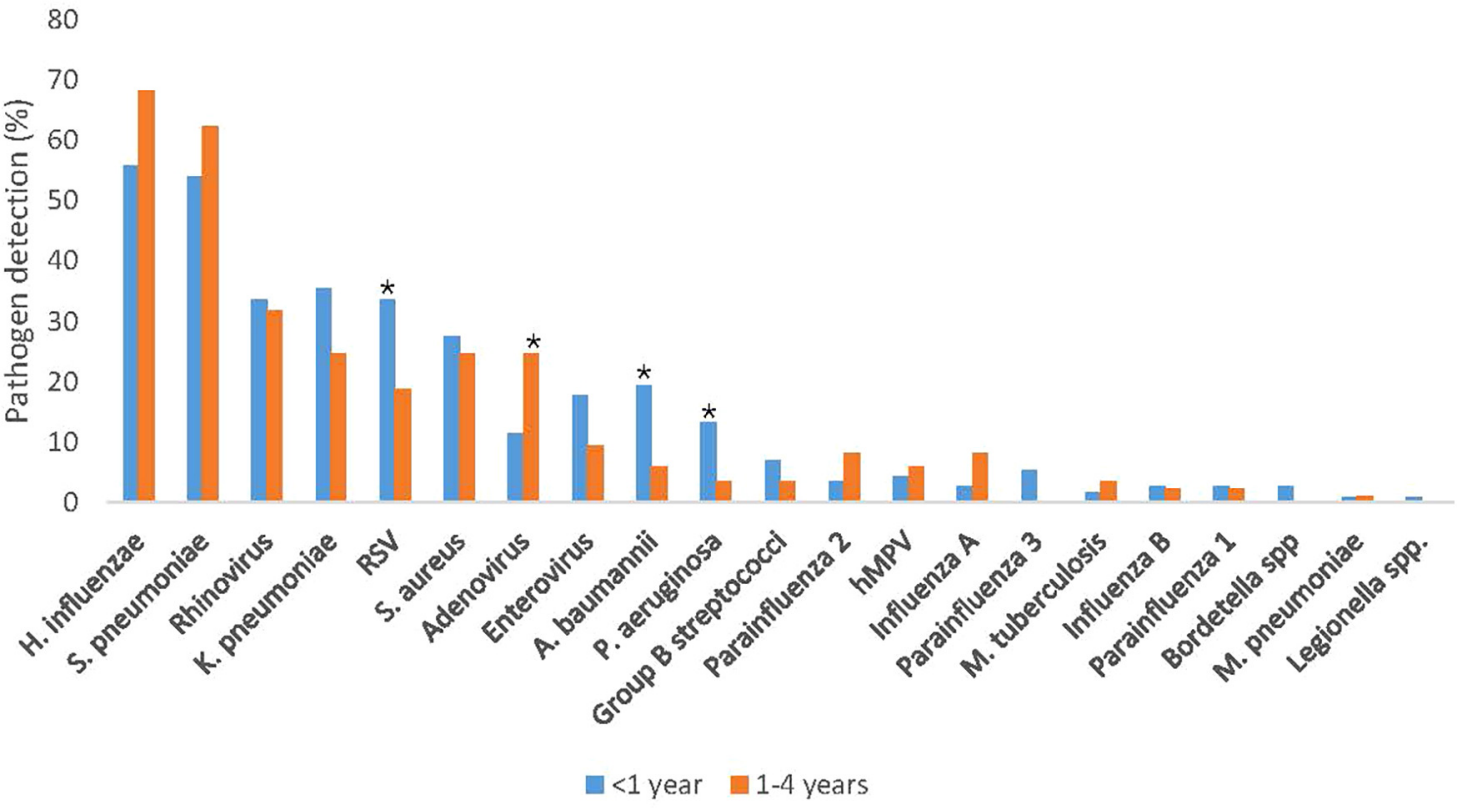
Pathogen detection rate stratified by age, among children aged <5 years hospitalized with severe respiratory illness, January–December 2017 (*N* = 198). RSV, respiratory syncytial virus. *P < 0.05 considered statistically significant.

Comparing pathogen prevalence between the infants aged <1 year and the children aged 1–4 years, RSV [34% [38/113] vs. 19% [16/85]; *p* = 0.031], *A. baumannii* [19% [22/113] vs. 6% [5/85]; *p* = 0.011], and *P. aeruginosa* [13% [15/113] vs. 4% [3/85]; *p* = 0.035] were more commonly detected among infants (Figure 1; Table 3). Further, PIV3 (6/113; 5%) and *Bordetella* spp. (3/113; 3%) were only detected in infants. Adenovirus was more prevalent among the older children [25% [21/85] vs. 12% [13/113]; *p* = 0.025] and was also more commonly detected in sputum compared to NPA [36% [16/44] vs. 11% [20/183]; *p* < 0.001] (Tables 2, 3).

**Table 3:** Prevalence of bacterial and viral pathogens, stratified by age group, among young children aged <5 years hospitalized with severe respiratory illness, January–December 2017.

Pathogen	All patients; *n* (%)	Age; *n* (%)		*p*-value
	*N* = 198	<1 year (*N* = 113)	1–4 years (*N* = 85)	
Any pathogen	189 (95)	111 (98)	78 (92)	0.255
Bacteria				
Any bacteria	177 (89)	106 (94)	71 (84)	**0**.**037**
*H. influenzae*	121 (61)	63 (56)	58 (68)	0.102
*S. pneumoniae*	114 (58)	61 (54)	53 (62)	0.301
*K. pneumoniae*	61 (31)	40 (35)	21 (25)	0.145
*S. aureus*	52 (26)	31 (27)	21 (25)	0.788
*A. baumannii*	27 (14)	22 (19)	5 (6)	**0**.**011**
*P. aeruginosa*	18 (9)	15 (13)	3 (4)	**0**.**035**
Group B streptococci	11 (6)	8 (7)	3 (4)	0.444
*M. tuberculosis*	5 (3)	2 (2)	3 (4)	0.405
*Bordetella* spp.	3 (2)	3 (3)	0	N/A
*M. pneumoniae*	2 (1)	1 (1)	1 (1)	0.252
*Legionella* spp.	1 (1)	1 (1)	0	N/A
Viruses				
Any virus	156 (79)	91 (81)	65 (76)	0.606
Rhinovirus	65 (33)	38 (34)	27 (32)	0.902
RSV	54 (27)	38 (34)	16 (19)	**0**.**031**
Adenovirus	34 (17)	13 (12)	21 (25)	**0**.**025**
Enterovirus	28 (14)	20 (18)	8 (9)	0.147
Parainfluenza 2	11 (6)	4 (4)	7 (8)	0.265
hMPV	10 (5)	5 (4)	5 (6)	0.892
Influenza A	10 (5)	3 (3)	7 (8)	0.148
Parainfluenza 3	6 (3)	6 (5)	0	N/A
Influenza B	5 (3)	3 (3)	2 (2)	1.000
Parainfluenza 1	5 (3)	3 (3)	2 (2)	1.000
Co-detections				
≥2 pathogens	174 (88)	106 (94)	68 (80)	**0**.**006**
≥2 bacteria	142 (72)	82 (73)	60 (71)	0.883
≥2 viruses	64 (32)	36 (32)	28 (33)	0.994
Any bacteria + any virus	144 (73)	86 (76)	58 (68)	0.285
Any bacteria only	33 (17)	20 (18)	13 (15)	0.797
Any virus only	12 (6)	5 (4)	7 (8)	0.417

RSV, respiratory syncytial virus; hMPV, human metapneumovirus; N/A, not applicable. *P* < 0.05 considered statistically significant when comparing pathogen detection stratified by age group.

Co-detections of ≥2 pathogens were common (174/198; 88%) and were more prevalent among infants compared to older children [94% [106/113] vs. 80% [68/85]; *p* = 0.006] (Table 3). Most co-detections were bacterial-viral (144/198, 73%) while single viral infections were rare (12/198, 6%). Common bacterial pathogens that were detected as a co-pathogen with a virus were *H. influenzae* (102/144, 71%), *S. pneumoniae* (100/144, 69%), *K. pneumoniae* (43/144, 30%), *S. aureus* (41/144, 28%), and *A. baumannii (*20/144, 14%) as was noted in one of the two cases that died. We detected *A. baumannii, S. pneumoniae, K. pneumoniae, H. influenzae, P. aeruginosa, S. aureus* and human parainfluenza type 3 in a 6-month-old decedent, and only bacteria (*S. pneumoniae, K. pneumoniae, P. aeruginosa,* and *S. aureus*) in the second deceased patient (aged 11 months).

The majority (129/198; 65%) of the specimens were collected in the autumn (75/198; 38%) and winter months (54/198; 27%) (Tables 1; Supplementary Table S2). However, overall pathogen detection was the same throughout the seasons (Supplementary Table S2). At individual pathogen level, *S. pneumoniae* and *H. influenzae* were more prevalent in winter months, each at 70% (38/54), compared to the other seasons, albeit not statistically significant (*p* = 0.136 and 0.263, respectively). RSV, on the other hand, was more common in the summer (19/49; 39%) and autumn months (22/75; 29%) compared to the other seasons (*p* < 001).

## 4 Discussion

This study reports on the detection of respiratory pathogens, using a multi-pathogen PCR assay, among children aged <5 years hospitalized with SRI in South Africa in 2017. A potential respiratory pathogen was detected in 95% of cases: leading viruses included rhinovirus, RSV, adenovirus, and enteroviruses while the leading bacteria, in both NPA and induced sputum, included *H. influenzae*, *S. pneumoniae* and *K. pneumoniae* as per previous reports (4, 15–19). RSV, *A. baumannii*, and *P. aeruginosa* were more prevalent among infants compared to children aged 1–4 years. *Bordetella* spp. and PIV3 were also only detected in infants but the detection rates were low owing to our small sample size and thus hindering the ability to accurately identify any differences between the age groups.

The high prevalence of RSV (27%) among children aged <5 years hospitalized with SRI is well documented (18, 20). Pretorius et al. previously reported a 25% prevalence of RSV among children aged <5 years hospitalized with severe acute respiratory illness in South Africa between 2012 and 2015 (18). Similar findings for RSV in this age group were also reported in Niger in 2015 (23%) (16), and in the Pneumonia Etiology Research for Child Health (PERCH) study (20%–40%) in The Gambia, Mali, Kenya, Zambia, South Arica, Bangladesh and Thailand between 2011 and 2014 (15). Further, our findings that the prevalence of RSV infection was higher among infants than older children is supported by findings from the PERCH study which reported a RSV prevalence of 40% in this age group compared to 20% in children aged 1–4 years (15). Moyes et al. estimated a mean annual total of 96,220 (95% CI 66,470–132,844) cases of RSV-associated SRI among young children aged <5 years in South Africa between 2011 and 2016. During this period, the burden of RSV disease was the highest among the <1-month age group with RSV-associated deaths highest in the first and second month of life (20). Recently, the FDA has licensed two products which prevent RSV disease in infants, a maternal vaccine and a long-acting monoclonal antibody prophylactic (21). Baral et al. estimated that RSV maternal immunisation has the potential to prevent about 28% to 31% of RSV cases, and 40% to 44% of RSV hospitalizations (22). While a number of these RSV maternal immunisation trials are ongoing, Kampmann et al. reported on the high efficacy of the bivalent RSV prefusion F protein–based (RSVpreF) vaccine administered through maternal immunisation in the 24–36 weeks gestation period; the vaccine was efficacious against severe medically-attended RSV-associated lower respiratory tract illness in the first 3 months of life (23). Further, Mahtab et al. recently identified RSV among the leading pathogens in deaths due to pneumonia in sub-Saharan African and South Asia between 2016 and 2022, particularly in the first 6 months of life (24). These and our findings highlight the urgency for continued efforts towards preventative public health interventions to reduce the burden of RSV-associated SRI, particularly in the first year of life and in low- and middle-income countries where the burden is highest.

In our study, a bacterial pathogen was detected in 89% of the cases and were more prevalent amongst infants compared to children aged 1–4 years. *Streptococcus pneumoniae* and *H. influenzae* were the most commonly detected at 58% and 61% prevalence, respectively. This is similar to a previous report from South Africa by Zar et al. (6). In their study, however, they reported similar detection rates between cases and healthy controls thus attributing the high detection rates to carriage.

Pathogen co-detections were common with bacterial and viral co-infections occurring the most frequently, as reported elsewhere (6, 25). Approximately 74% and 70% of cases testing positive for *A. baumannii* and *K. pneumoniae*, respectively, were also positive for a virus. Similarly, *S. pneumoniae* and *H. influenzae* were co-detected with a virus in 88% and 84% of the cases, respectively. Viral and bacterial co-infections increase the risk of severe disease (26, 27); single infections with a virus were rare (6%) among the SRI cases in our study.

In South Africa, the administration of empiric antibiotic therapy depends on the child's age, among other factors, and can include amoxicillin-clavulanate, ampicillin and gentamicin (8). In our study, 96% of the children received antibiotics on admission; the low prevalence of virus-only infections and high prevalence of bacterial detections whether as a single or a co-pathogen highlight the importance of investigating the interplay between viruses and colonising bacteria in the pathogenesis of SRI.

The main limitation of our study is the lack of healthy controls which would have been useful to determine the etiological fraction of individual pathogens to disease. Studies have reported high carriage rates of *S. pneumoniae*, *S. aureus, K. pneumoniae, H. influenzae, A. baumannii,* and *P. aeruginosa* in the upper respiratory tract, particularly in healthy young children (6, 15, 28–31). Testing of upper respiratory tract samples only may not, therefore, discriminate between colonizing and pathogenic organisms, making it difficult to attribute etiology. Future studies can apply pathogen-specific density thresholds in upper respiratory tract specimens when establishing SRI etiology as some studies have shown a higher median density for some bacteria in cases compared to healthy controls (32, 33). Also, the lack of data from analysis of invasive clinical specimens also limits our ability to discriminate between colonizing and pathogenic organisms as the clinical utility of testing respiratory specimens is in the detection of viruses and atypical pneumonia pathogens. However, viruses including adenovirus and rhinovirus have been detected amongst healthy controls (15) and therefore their roles in disease could not be established. Some case-control studies have shown association of rhinoviruses and enteroviruses with SRI whereas others, such as adenovirus and parainfluenza viruses type 1–3 have shown weak or no attribution to disease (6, 15, 18). Pathogen co-detections were common with bacterial and viral co-infections occurring the most frequently. Primary infection with a virus predisposes an individual to a secondary bacterial infection however, with our study design, we were not able to determine whether one pathogen preceded the other. We were unable to determine seasonality of individual pathogens over longer periods of time as our study period was only one year, we could however describe the prevalence of each pathogen during each season in the one year of the study. Lastly, we do not have data on the number of SRI cases presenting at the study sites and our small sample size only represents 55% of the cases enrolled at the study sites and may therefore not reflect the true distribution of pathogens in these settings and thus limiting our ability to generalize our findings.

## 5 Conclusion

In our study we show a disproportionately higher number of SRI cases in infants aged <1 year compared to children aged 1–4 years. The rate of bacterial detections was high particularly among infants compared to the children aged 1–4 years. RSV, *A. baumannii*, and *P. aeruginosa* were more commonly identified in infants compared to older children, and the majority of the cases tested positive for more than one pathogen of which bacterial-viral co-detections occurred in 73% of all cases tested. The high rate of co-detections requires clinical evaluation to determine their relevance for individual treatment and management decisions. Given the high prevalence of RSV amongst children hospitalized with SRI, particularly in the first year of life, there is an urgent need for continued efforts towards early-life RSV prevention strategies to help reduce RSV-associated morbidity and mortality. These include access to maternal vaccines and monoclonal antibodies particularly in low- and middle-income countries which experience the highest burden of RSV-associated disease.

## Data Availability

The original contributions presented in the study are included in the article/Supplementary Material, further inquiries can be directed to the corresponding author.
