# Supplementary material for: Respiratory pathogens detected in children aged <5 years hospitalized with severe respiratory illness, South Africa, 2017
Source: Front Pediatr. 2025 Jun 27;13:1498197. doi: 10.3389/fped.2025.1498197 (PMC12245838; doi:10.3389/fped.2025.1498197)
Supplement: Supplementary file 1 [file Table1.docx]

Supplementary Material

**Table S1:** Detection of bacterial pathogens in paired sputum and NPA of patients hospitalized with SRI, N=29

| **Pathogen** | **Total positive on sputum** | **Sputum + NPA positive (%)** | **Sputum positive only** |
| --- | --- | --- | --- |
| *H. influenzae* | 23 | 21 (91) | 2 |
| *S. pneumoniae* | 23 | 19 (82) | 4 |
| *K. pneumoniae* | 7 | 4 (57) | 3 |
| *S. aureus* | 5 | 2 (40) | 3 |
| *A. baumannii* | 5 | 1 (20) | 4 |
| *P. aeruginosa* | 2 | 0 | 2 |
| GBS | 1 | 0 | 1 |
| *M. tuberculosis* | 2 | 0 | 2 |
| *Bordetella* spp. | 1 | 0 | 1 |

NPA, nasopharyngeal aspirate. GBS, Group B streptococci.

**Table S2:** Seasonal frequency of common bacterial and viral pathogens among children aged <5 years hospitalized with severe respiratory illness, January – December 2017

| **Pathogen** | **All patients; n (%)** | **Season; n (%)** | | | | **p-value** |
| --- | --- | --- | --- | --- | --- | --- |
|  | **N=198** | **Summer (N=49)** | **Autumn (N=75)** | **Winter (N=54)** | **Spring (N=20)** |  |
| Any pathogen | 189 (95) | 47 (96) | 70 (93) | 53 (98) | 19 (95) | 0.634 |
| **Bacteria** |  |  |  |  |  |  |
| Any bacteria | 177 (89) | 44 (90) | 66 (88) | 50 (93) | 17 (85) | 0.764 |
| *H. influenzae* | 121 (61) | 30 (61) | 40 (53) | 38 (70) | 13 (65) | 0.263 |
| *S. pneumoniae* | 114 (58) | 26 (53) | 38 (51) | 38 (70) | 12 (60) | 0.136 |
| *K. pneumoniae* | 61 (31) | 20 (41) | 24 (32) | 11 (20) | 6 (30) | 0.163 |
| *S. aureus* | 52 (26) | 7 (14) | 23 (31) | 17 (31) | 5 (25) | 0.161 |
| *A. baumannii* | 27 (14) | 14 (29) | 8 (11) | 4 (7) | 1 (5) | **0.005** |
| **Viruses** |  |  |  |  |  |  |
| Any virus | 156 (79) | 41 (84) | 57 (76) | 44 (81) | 14 (70) | 0.531 |
| Rhinovirus | 65 (33) | 13 (27) | 29 (39) | 19 (35) | 4 (20) | 0.321 |
| RSV | 54 (27) | 19 (39) | 22 (29) | 13 (24) | 0 | **<0.001** |
| Adenovirus | 34 (17) | 9 (18) | 11 (15) | 9 (17) | 5 (25) | 0.741 |
| Enterovirus | 28 (14) | 8 (16) | 11 (15) | 8 (15) | 1 (5) | 0.633 |

RSV, respiratory syncytial virus. N/A, not applicable. P <0.05 considered statistically significant. *Seasons were defined as follows: summer, Dec/Jan/Feb; autumn, Mar/Apr/May; winter, Jun/Jul/Aug; spring, Sep/Oct/Nov
